# Supplementary material for: The influence of language proficiency, acculturation stress, and institutional support in enhancing personal development of international students in China
Source: PLoS One. 2025 Apr 4;20(4):e0315040. doi: 10.1371/journal.pone.0315040 (PMC11970667; doi:10.1371/journal.pone.0315040)
Supplement: S1 File — (DOCX) [file pone.0315040.s001.docx]

**Appendix A: Demographic information**

Question 1: Gender

| Options | **Subtotal** | **Proportion** |
| --- | --- | --- |
| Male. | 256 | 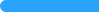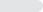70.14% |
| Female. | 103 | 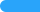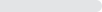28.22% |
| Prefer not to say. | 6 | 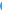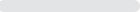1.64% |
| Number of valid entries for this question | 365 |  |

Question 2: Age

| **Options** | **Subtotal** | **Proportion** |
| --- | --- | --- |
| Less than 20 | 22 | 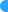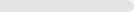6.03% |
| 20-30 | 210 | 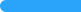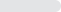57.53% |
| 30-40 | 123 | 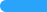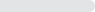33.7% |
| More than 40 | 10 | 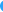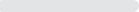2.74% |
| Number of valid entries for this question | 365 |  |

Question 3: Marital Status

| **Options** | **Subtotal** | **Proportion** |
| --- | --- | --- |
| Married. | 118 | 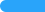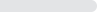32.33% |
| Unmarried. | 240 | 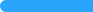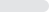65.75% |
| Divorced. | 7 | 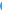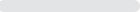1.92% |
| Number of valid entries for this question | 365 |  |

Question 4: Cultural Background

| **Options** | **Subtotal** | **Proportion** |
| --- | --- | --- |
| Asian. | 272 | 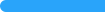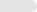74.52% |
| European. | 12 | 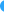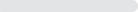3.29% |
| African | 53 | 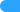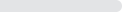14.52% |
| American | 7 | 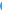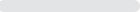1.92% |
| Other | 21 | 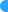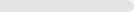5.75% |
| Number of valid entries for this question | 365 |  |

Question 5: Academic Level

| **Options** | **Subtotal** | **Proportion** |
| --- | --- | --- |
| Bachelor's | 124 | 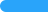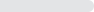33.97% |
| Master's. | 113 | 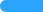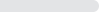30.96% |
| PhD. | 122 | 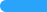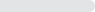33.42% |
| Postdoc. | 6 | 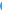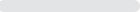1.64% |
| Number of valid entries for this question | 365 |  |

Question 6: Chinese Language Proficiency

| **Options** | **Subtotal** | **Proportion** |
| --- | --- | --- |
| Beginner. | 232 | 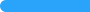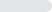63.56% |
| Intermediate. | 102 | 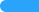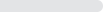27.95% |
| Advanced. | 25 | 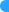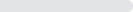6.85% |
| (Null) | 6 | 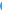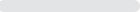1.64% |
| Number of valid entries for this question | 365 |  |

Question 7: Monthly Consumption (in RMB)

| **Options** | **Subtotal** | **Proportion** |
| --- | --- | --- |
| less than 2000 | 123 | 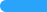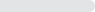33.7% |
| 2000-3000 | 133 | 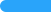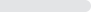36.44% |
| 3000-4000 | 81 | 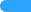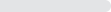22.19% |
| 4000 and above | 24 | 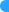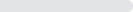6.58% |
| (Null) | 4 | 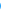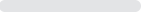1.1% |
| Number of valid entries for this question | 365 |  |

Question 8: Source of Funding

| **Options** | **Subtotal** | **Proportion** |
| --- | --- | --- |
| Family/Self. | 136 | 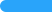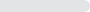37.26% |
| Scholarship from home government. | 33 | 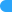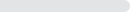9.04% |
| Chinese scholarship (CSC, University. etc). | 196 | 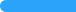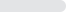53.7% |
| Number of valid entries for this question | 365 |  |

Question 9: Daily Reading Time

| **Options** | **Subtotal** | **Proportion** |
| --- | --- | --- |
| Less than 2 hours | 60 | 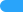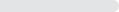16.44% |
| 2-4 hours | 116 | 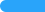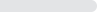31.78% |
| 4-8 hours. | 127 | 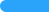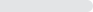34.79% |
| More than 8 hours | 60 | 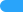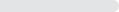16.44% |
| (Null) | 2 | 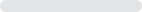0.55% |
| Number of valid entries for this question | 365 |  |

Question 10: Relationship with Professors

| **Options** | **Subtotal** | **Proportion** |
| --- | --- | --- |
| Excellent | 205 | 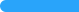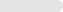56.16% |
| Somewhat Good | 74 | 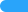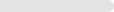20.27% |
| Average | 74 | 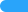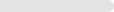20.27% |
| Not Good | 10 | 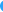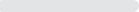2.74% |
| Very Bad | 2 | 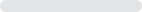0.55% |
| Number of valid entries for this question | 365 |  |

Question 11: Describe the extent of your interaction with local students within your research group

| **Options** | **Subtotal** | **Proportion** |
| --- | --- | --- |
| Regular collaboration on projects | 113 | 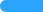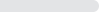30.96% |
| Casual discussions and knowledge exchange | 142 | 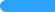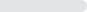38.9% |
| Limited interaction | 109 | 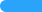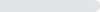29.86% |
| (Null) | 1 | 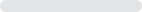0.27% |
| Number of valid entries for this question | 365 |  |

Question 12: How frequently do you actively participate in seminars organized by your research group?

| **Options** | **Subtotal** | **Proportion** |
| --- | --- | --- |
| Daily | 37 | 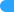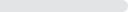10.14% |
| Weekly | 158 | 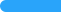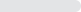43.29% |
| Monthly | 165 | 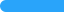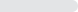45.21% |
| (Null) | 5 | 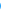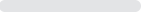1.37% |
| Number of valid entries for this question | 365 |  |

Question 13: How have you actively engaged with professionals from diverse backgrounds in China during your academic pursuits?

| **Options** | **Subtotal** | **Proportion** |
| --- | --- | --- |
| Attending conferences and workshops | 178 | 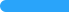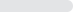48.77% |
| Networking events | 106 | 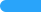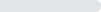29.04% |
| Collaborative projects | 67 | 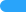18.36% |
| (Null) | 14 | 3.84% |
| Number of valid entries for this question | 365 |  |

Question 14: What measures do you take to support the integration of international and local students within the research group?

| **Options** | **Subtotal** | **Proportion** |
| --- | --- | --- |
| Mentorship programs | 100 | 27.4% |
| Cultural exchange initiatives | 161 | 44.11% |
| Inclusive communication practices | 97 | 26.58% |
| (Null) | 7 | 1.92% |
| Number of valid entries for this question | 365 |  |

Question 15: Frequency of Engagement with Local Community

| **Options** | **Subtotal** | **Proportion** |
| --- | --- | --- |
| Rarely. | 125 | 34.25% |
| Seldom. | 121 | 33.15% |
| Frequently. | 114 | 31.23% |
| (Null) | 5 | 1.37% |
| Number of valid entries for this question | 365 |  |

Question 16: Learning Environment Preference

| **Options** | **Subtotal** | **Proportion** |
| --- | --- | --- |
| Traditional Classroom. | 120 | 32.88% |
| Interactive and Collaborative. | 201 | 55.07% |
| Online | 41 | 11.23% |
| (Null) | 3 | 0.82% |
| Number of valid entries for this question | 365 |  |

Question 17: Coping Mechanisms

| **Options** | **Subtotal** | **Proportion** |
| --- | --- | --- |
| Exercise. | 146 | 40% |
| Meditation. | 58 | 15.89% |
| Socializing. | 128 | 35.07% |
| Seeking Counseling. | 33 | 9.04% |
| Number of valid entries for this question | 365 |  |

Question 18: Plans to Continue Education/Work in China after Current Degree

| **Options** | **Subtotal** | **Proportion** |
| --- | --- | --- |
| Yes | 273 | 74.79% |
| NO | 92 | 25.21% |
| Number of valid entries for this question | 365 |  |
