# Supplementary material for: The influence of language proficiency, acculturation stress, and institutional support in enhancing personal development of international students in China
Source: PLoS One. 2025 Apr 4;20(4):e0315040. doi: 10.1371/journal.pone.0315040 (PMC11970667; doi:10.1371/journal.pone.0315040)
Supplement: Attachment — Submitted filename: PONE-D-24-35389_reviewer.pdf [file pone.0315040.s004.pdf]

PLOS ONE

Exploring the Relationship of Diversity Experiences, Innovative Learning Environments, and Personal Development among International Students in China

--Manuscript Draft--

**Manuscript Number:**	PONE-D-24-35389
**Article Type:**	Research Article
**Full Title:**	Exploring the Relationship of Diversity Experiences, Innovative Learning Environments, and Personal Development among International Students in China
**Short Title:**	Relationship of Diversity Experiences, Innovative Learning Environments, and Personal Development
**Corresponding Author:**	Muhammad Azram, Ph.D<br>Xi'an Jiaotong University<br>Xi'an, None Selected CHINA
**Keywords:**	Diversity experiences; Innovative learning environments; Language Proficiency; Acculturation Stress; Institutional Support; Personal development; International students
**Abstract:**	This paper explores the intricate relationship between diversity experiences and innovative learning environments in Higher Education Institutions (HEIs) in Shaanxi, China, and their impact on student personal development. Using a quantitative research design, 364 participants from various HEIs were selected through convenience sampling, with data collected via a questionnaire on the Questionnaire Star platform. Structural Equation Modeling (SEM) using Smart PLS4 and descriptive statistics revealed a positive association between diversity experiences and personal development. However, innovative learning environments and personal development were not found to influence each other directly. The study identified a weak correlation between acculturation stress and diversity experiences but a significant correlation between diversity experiences and language proficiency. Innovative learning environments showed positive associations with both acculturation stress and language proficiency, while language proficiency and acculturation stress were positively correlated with personal development. Language proficiency indirectly influences human development through diverse experiences and innovative learning settings. The study highlighted that the association between diversity experiences and personal growth is strengthened by institutional support, providing valuable insights into factors influencing international students' personal development.
**Order of Authors:**	Muhammad Azram, Ph.D
	MEI Hong
	Waqar Ahmad
**Additional Information:**	
**Question**	**Response**
**Financial Disclosure**<br><br>Enter a financial disclosure statement that describes the sources of funding for the work included in this submission. Review the submission guidelines for detailed requirements. View published research articles from *PLOS ONE* for specific examples.<br><br>This statement is required for submission	The author(s) received no specific funding for this work.

Powered by Editorial Manager® and ProduXion Manager® from Aries Systems Corporation

and **will appear in the published article** if the submission is accepted. Please make sure it is accurate.<br><br>**Funded studies**<br>Enter a statement with the following details:<br>• Initials of the authors who received each award<br>• Grant numbers awarded to each author<br>• The full name of each funder<br>• URL of each funder website<br>• Did the sponsors or funders play any role in the study design, data collection and analysis, decision to publish, or preparation of the manuscript?<br><br>Did you receive funding for this work?	
**Competing Interests**<br><br>Use the instructions below to enter a competing interest statement for this submission. On behalf of all authors, disclose any competing interests that could be perceived to bias this work—acknowledging all financial support and any other relevant financial or non-financial competing interests.<br><br>This statement is required for submission and **will appear in the published article** if the submission is accepted. Please make sure it is accurate and that any funding sources listed in your Funding Information later in the submission form are also declared in your Financial Disclosure statement.<br><br>View published research articles from *PLOS ONE* for specific examples.	NO authors have competing interests

**NO authors have competing interests**<br><br>Enter: *The authors have declared that no competing interests exist.*<br><br>**Authors with competing interests**<br><br>Enter competing interest details beginning with this statement:<br><br>*I have read the journal's policy and the authors of this manuscript have the following competing interests: [insert competing interests here]*<br><br>* typeset	
**Ethics Statement**<br><br>Enter an ethics statement for this submission. This statement is required if the study involved:<br><br>• Human participants<br>• Human specimens or tissue<br>• Vertebrate animals or cephalopods<br>• Vertebrate embryos or tissues<br>• Field research<br><br>Write "N/A" if the submission does not require an ethics statement.<br><br>General guidance is provided below. Consult the submission guidelines for detailed instructions. **Make sure that all information entered here is included in the Methods section of the manuscript.**	The approval from the Institutional Review Board (IRB) or Ethics Committee has been obtained and the relevant document has been uploaded as part of this submission.

Powered by Editorial Manager® and ProduXion Manager® from Aries Systems Corporation

**Format for specific study types**<br><br>**Human Subject Research (involving human participants and/or tissue)**<br>• Give the name of the institutional review board or ethics committee that approved the study<br>• Include the approval number and/or a statement indicating approval of this research<br>• Indicate the form of consent obtained (written/oral) or the reason that consent was not obtained (e.g. the data were analyzed anonymously)<br><br>**Animal Research (involving vertebrate animals, embryos or tissues)**<br>• Provide the name of the Institutional Animal Care and Use Committee (IACUC) or other relevant ethics board that reviewed the study protocol, and indicate whether they approved this research or granted a formal waiver of ethical approval<br>• Include an approval number if one was obtained<br>• If the study involved *non-human primates*, add *additional details* about animal welfare and steps taken to ameliorate suffering<br>• If anesthesia, euthanasia, or any kind of animal sacrifice is part of the study, include briefly which substances and/or methods were applied<br><br>**Field Research**<br><br>Include the following details if this study involves the collection of plant, animal, or other materials from a natural setting:<br>• Field permit number<br>• Name of the institution or relevant body that granted permission	
**Data Availability**<br><br>Authors are required to make all data underlying the findings described fully available, without restriction, and from the time of publication. PLOS allows rare exceptions to address legal and ethical concerns. See the PLOS Data Policy and FAQ for detailed information.	No - some restrictions will apply

A Data Availability Statement describing where the data can be found is required at submission. Your answers to this question constitute the Data Availability Statement and **will be published in the article**, if accepted.<br><br>**Important:** Stating 'data available on request from the author' is not sufficient. If your data are only available upon request, select 'No' for the first question and explain your exceptional situation in the text box.<br><br>Do the authors confirm that all data underlying the findings described in their manuscript are fully available without restriction?	
**Describe where the data may be found in full sentences. If you are copying our sample text, replace any instances of XXX with the appropriate details.**<br><br>• If the data are **held or will be held in a public repository**, include URLs, accession numbers or DOIs. If this information will only be available after acceptance, indicate this by ticking the box below. For example: *All XXX files are available from the XXX database (accession number(s) XXX, XXX.*).<br>• If the data are all contained **within the manuscript and/or Supporting Information files**, enter the following: *All relevant data are within the manuscript and its Supporting Information files.*<br>• If neither of these applies but you are able to provide **details of access elsewhere**, with or without limitations, please do so. For example:<br><br>*Data cannot be shared publicly because of [XXX]. Data are available from the XXX Institutional Data Access / Ethics Committee (contact via XXX) for researchers who meet the criteria for access to confidential data.*<br><br>*The data underlying the results presented in the study are available from (include the name of the third party*	All relevant data are within the manuscript and its Supporting Information

and contact information or URL).<br>• This text is appropriate if the data are owned by a third party and authors do not have permission to share the data.<br><br>* typeset	
Additional data availability information:	

Click here to access/download;Manuscript;Manuscript.docx

Exploring the Relationship of Diversity Experiences, Innovative Learning Environments, and Personal Development among International Students in China

Muhammad Azram[1*], MEI Hong[1], Waqar Ahmad[2]

[1] *School of Public Policy and Administration, Xi'an Jiaotong University, Xi'an Shaanxi, China*

[2] *Department of Management Sciences, University of Central Punjab, Lahore, Pakistan*

[*] **Corresponding author**
Email: azram_khan@stu.xjtu.edu.cn (Muhammad Azram)

Exploring the Relationship of Diversity Experiences, Innovative Learning Environments, and Personal Development among International Students in China

Abstract

This paper explores the intricate relationship between diversity experiences and innovative learning environments in Higher Education Institutions (HEIs) in Shaanxi, China, and their impact on student personal development. Using a quantitative research design, 364 participants from various HEIs were selected through convenience sampling, with data collected via a questionnaire on the Questionnaire Star platform. Structural Equation Modeling (SEM) using Smart PLS4 and descriptive statistics revealed a positive association between diversity experiences and personal development. However, innovative learning environments and personal development were not found to influence each other directly. The study identified a weak correlation between acculturation stress and diversity experiences but a significant correlation between diversity experiences and language proficiency. Innovative learning environments showed positive associations with both acculturation stress and language proficiency, while language proficiency and acculturation stress were positively correlated with personal development. Language proficiency indirectly influences human development through diverse experiences and innovative learning settings. The study highlighted that the association between diversity experiences and personal growth is strengthened by institutional support, providing valuable insights into factors influencing international students' personal development.

Keywords: Diversity experiences; Innovative learning environments; Language Proficiency; Acculturation Stress; Institutional Support; Personal development; International students

Introduction

The impact of diverse experiences and innovative learning environments on students' personal growth is becoming increasingly apparent in today's worldwide environment. Students interact with people from various backgrounds, languages, and cultures. They also have access to opportunities to enhance their language skills, reduce acculturation stress, and spend time with people with a broader worldview. The current study looks at the relationship between innovative learning environments, diverse experiences, and the personal growth of international students. Additionally, a focus should be placed on the moderating influence of institutional support and the mediating roles of language proficiency and acculturation stress.

Numerous studies have shown that students' personal development is positively influenced by their positive exposure to other cultures and languages (Bernard, Borokhovski et al. 2019). The advantages of creative learning spaces, which can foster creativity, critical thinking, and teamwork while advancing students' social and cognitive development, are emphasized by Souto-Manning, Falk et al. (2019). It is essential to keep in mind that the impact of these settings and experiences on students' personal growth may depend on their language skills as well as their capacity to handle the stress of acculturation during the duration of the study (Charbonneau, deLeyer-Tiarks et al. 2022). Assistance from educational institutions is crucial for encouraging students to grow personally in various innovative learning environments (van den Berg, Brouwer, et al. 2020, Dilara and YILMAZ 2021).

To offer practical suggestions for educators and decision-makers, this study will examine the complex relationship between these variables by growing more conscious of the connections among diverse learning experiences, innovative learning environments, and personal

development. We know how to make classrooms more hospitable and supportive of students from different backgrounds. These understandings can guide the development of effective educational methods and policies that support students' personal development. This study aims to assist ongoing initiatives in education aimed at providing balanced and enjoyable learning environments for students.

Diversity experiences refer to students' interactions and encounter with individuals from different cultural, ethnic, and social backgrounds (do Amaral, Romani-Dias et al. 2022). These experiences can be important in shaping the students' personal development. Several investigations examined the diversity experiences impact on students' personal growth. For instance, exposure to diverse environments directed to increased intercultural competence and adaptability in students (Onosu 2021). The study underscored that diversity experiences, whether within one's own country or through global interactions, contribute to personal growth and the development of intercultural skills (Zylkiewicz-Plonska and Acienė 2014).

Diversity experiences expose students to different perspectives, values, and ways of thinking (Onosu 2021). This experience supports broadening their understanding of other cultures and challenges. It enables students to develop empathy, cultural sensitivity, and the ability to direct complex intercultural interactions (Üzar-Özçetin, Çelik, et al. 2022). Furthermore, diversity experiences improve the development of essential skills such as collaboration, communication, and problem-solving in multicultural contexts (Simons et al., 2023). Students learn to indicate the challenges and difficulties that arise from cultural differences, equipping them with valuable skills that are highly sought after in today's globalized workforce (Simons, Schoneman, et al., 2023).

In education, innovative learning environments include contemporary and technologically advanced teaching methods and settings (Shaqour, 2021). Extensive investigations have shown that these environments positively affect students' academic and personal development. They endorse the significance of critical thinking and problem-solving skills (Yulianti & Badruzsaufari, 2020). Using technology and interactive learning tools, students can actively participate in collaborative activities and investigate real-world scenarios (Melo, Luft, et al. 2021).

Innovative learning environments foster collaboration and creativity among students by contributing opportunities for interdisciplinary approaches and project-based learning. Additionally, for these advantages, such environments also embrace technology integration to enhance learning. Research has always supported the effectiveness of technology and integration in engaging students and facilitating their comprehension of multifaceted concepts. Computer-assisted language learning and online resources, such tools enable personalized learning experiences. Students with additional resources beyond traditional textbooks (Tawafak, Malik, et al. 2021).

Language proficiency is “the level of skill and competence in a new language” (Supeni and Fauziah 2020). It involves understanding, listening, speaking, reading, and writing in the target language. Having strong language proficiency enables individuals with people from different cultures to communicate and interact (Novita and Purwati 2021). A high level of language proficiency can contribute to successful adjustment and intercultural effectiveness, whether for personal or professional reasons. (Yang 2020). A person's language proficiency is determined by their ability to understand the language in different contexts. Students often face

language barriers, especially when they study abroad (Law and Liu, 2023).

Acculturation stress is “the psychological discomfort and challenges experienced when adapting to a new culture” (Koschollek, Kajikhina, et al., 2022). Research indicates that the stress due to adapting to a new culture can impede personal development. They understand how this stress affects a student's growth and well-being (Gebregergis 2018). However, different things can interfere with the student's adjustment, which can cause acculturative stress (Machul, Bieniak, Mental health can badly be affected by acculturative stress, such as depression, bulimia symptoms, and problems with the body. Academic institutions and mental health practitioners must understand the variables linked to acculturative stress within this particular era (Edara 2018). By recognizing these elements, suitable interventions and support services can be implemented to help students overcome acculturation difficulties (Abu-Kaf and Khalaf, 2020).

Institutional support is “the resources and assistance educational institutions offer their students. Researchers highlighted the value of institutional support in endorsing students' personal development (Stoszkowski and McCarthy 2018). This support includes programs for academic advice, cultural integration, and counseling services. The administrative detachments of the educational institutions, including financial assistance, admissions, payroll, public safety, athletics, food service, and registration, are also incorporated in the category of institutional support. These departments are vital to educational institutions' smooth and effective operation because they give students the support and resources required for their academic journey (Egarievwe 2021). The faculty, administration, and community funding agencies' support and the university's cooperation show that everyone is committed to the expansion and success of the student assistance program. These organizations guarantee that students receive thorough

support and access to resources that advance their academic and personal growth by matching their missions with the program's objectives (Glomo-Narzoles and Glomo-Palermo 2021). Institutional support is essential to facilitate students' growth and promote their success. (Mangus, Somers et al. 2021).

Hypotheses development between variables

This section explores hypotheses about how various variables impact international students' Personal Development (PD), based on existing literature and empirical studies. The researcher collectively suggests that Diversity Experiences (DE) can positively affect Personal Development (PD). Interactional diversity experiences positively affected the self-reported gains of white students and students of color (Herzog, 2022). Another study found that students with more experiences with diversity, such as enrollment in diversity courses and positive interactions with diverse peers, scored higher on academic self-confidence, social agency, and critical thinking disposition (Ahn, Kivlighan Jr et al. 2022). Finally, counseling students participating in personal growth groups as part of multicultural counseling courses experienced greater ethnic identity development. In this study, the relationship between diversity experiences and personal development which hypnotized as $\mathbf{H_1}$ is exist according to the literature.

Furthermore, the Prior researcher provides valuable insights regarding the interplay between Innovative Learning Environments (ILE) and individual personal development. An innovative educational environment influences a child's personal development (Marina, Nonna et al. 2018). The research focuses on professional development for innovative teaching and learning, highlighting its importance for personal growth and student achievement (Sancar, Atal, et al. 2021). The significance of cultural differences in handling tools like social networks and the

potential impact on academic development (Parkhouse, Lu et al. 2019). Overall, these studies suggest that innovative learning environments can contribute to personal development for students and educators. In this study, the relationship between innovative learning environments and personal development which hypnotized as $\mathbf{H_2}$ is exist according to this literature.

Moreover, studies collectively suggest that diversity experience can enhance Language Proficiency (LP). Individuals with international experience tend to have higher levels of open-mindedness, a key component of intercultural competence (Genkova 2016). The author further supported this by finding that students in diverse language classrooms were more likely to relate language learning to social justice issues (Kubota, Austin, The importance of cultural and linguistic competency in teachers is highlighted by two Researchers, who both emphasize the need for educators to model and support appropriate cultural competencies in increasingly diverse classrooms (Keengwe 2010, Zhou 2015). So, it can be hypothesized that $\mathbf{H_3}$ has considered as significant relationship exists between diversity experiences and language proficiency.

Many studies have explored the relationship between diversity experience and Acculturation Stress (AS). Language constraints and perceived cultural differences significantly contribute to acculturative stress among Chinese international students in the EU (Cao, Zhu et al. 2016). Social support and English proficiency are key factors in acculturative stress among international students in the US, with those from Asian countries and those who primarily socialized with non-Americans experiencing higher levels of stress (Poyrazli, Kavanaugh, et al. 2004). The role of personality traits and acculturation strategies in predicting acculturative stress among first-generation immigrants in Mauritius (Ramdhonee and Bhowon 2012). The

hypothesis **H_4** can be formulated as having a significant relationship exists between diversity experiences and acculturation stress.

Studies collectively suggest that Innovative learning environments and language proficiency can have a positive relationship. The role of technology in enhancing language skills, specifically noting the use of technology in an intercultural understanding course to improve English proficiency (Bruguier and Greathouse Amador 2012) (Banditvilai 2016). The author further claims this, emphasizing the value of experiential and digitally-mediated tools in language learning (Howard and Scott 2017). However, it is important to note that the impact of innovative learning environments on language proficiency can be influenced by cultural and contextual factors (Gabryś-Barker 2019). The hypothesis **H_5** can be formulated as having a significant relationship between innovative learning environments and language proficiency.

Additionally, innovative learning environments can significantly impact the acculturation stress experienced by international students. Students with broad social support and an integration approach to acculturation experienced lower stress levels (Sullivan and Kashubeck-West 2015). They further highlighted the role of language constraints and perceived cultural differences in influencing stressors, accounting for a significant portion of the variance in academic and social integration difficulties (Cao, Zhu et al. 2016). The author identified perceived discrimination, hate/rejection, fear, and cultural shock as key stressors, with perceived fear negatively impacting academic adjustment (Atteraya, 2021). The hypothesis **H_6** can be formulated as having a significant relationship between innovative learning environments and acculturation stress.

Besides, research has consistently shown a positive relationship between language proficiency and personal development. English proficiency development led to personal growth, including increased courage, enriched relationships, and positive attitudes (Chan and KAPONG 2021). The role of foreign language teaching in adult learners' personal development includes acquiring new personal qualities and pursuing self-improvement (Ariyan and Vadeeva 2019). Further, the connection between language proficiency and self-reported teaching ability was discussed, suggesting a potential link between language proficiency and pedagogical skills (Faez and Karas 2017). The hypothesis **H_7** can be formulated as having a significant relationship between language proficiency and personal development.

Furthermore, the studies collectively suggest that while acculturation stress can impact personal development, it is influenced by a range of individual and contextual factors. Certain dimensions of acculturation, such as self-control and collectivism, as well as personality traits, can predict personal growth and acculturation stress (Ramdhonee and Bhowon 2012). Further highlighted the role of social support and coping strategies in mitigating the adverse effects of acculturation stress on psychological functioning (Crockett, Iturbide et al. 2007). The hypothesis **H_8** can be formulated as having a significant relationship between acculturation stress and personal development.

Researchers collectively suggest that language proficiency can mediate between diversity experiences and personal development. Using language as a communicative tool in diverse contexts indicates its role in shaping personal experiences and interactions. Teachers and students can benefit from understanding language and its diversity to navigate intercultural challenges in higher education (Murray 2016). Investigation discussed how language

differences and second language acquisition experiences contributed to the development of identity in young Malaysian children (Yamat 2012). The hypothesis **H_9** can be formulated as language proficiency acting as a mediator between diversity experiences and personal development.

Moreover, studies indicate that language proficiency can play a mediating role between innovative learning environments and personal development. The importance of interactive learning technologies in developing students' foreign language communicative competence, which is seen as an integrative personal quality that mediates personal and professional development (Fahrutdinova, Yarmakeev, et al. 2014). Learners' perceptions of their language learning experiences highlight the role of digital learning profiles and the use of technologies in formal and informal contexts (Lintunen, Mutta et al. 2017). The investigation highlights the shift from teacher-centered to learner-centered environments, emphasizing the role of interactive communicative language tasks and the teacher as a facilitator of learning (Moeller and Catalano 2015). The hypothesis **H_{10}** can be formulated as language proficiency acting as a mediator between innovative learning environments and personal development.

(Katsiaficas, Suárez-Orozco, et al. 2013) suggested that acculturation stress can play a mediating role between diversity experiences and personal development. Perceptions of social support mediate the relationship between acculturative stress and internalizing symptoms in first-generation immigrant adolescents. Acculturation stress experienced by both multicultural adolescents and their parents negatively predicted the adolescents' career decisions, with career barriers mediating this relationship (Jo, Woo et al. 2023). The intercultural contact led to temporary phases of acculturative stress but also personal growth, suggesting a mediating role

of stress in the relationship between intercultural contact and self-esteem (Geeraert and Demoulin 2013). Different dimensions of acculturative stress mediated the association between acculturation and psychosocial functioning in Cuban American college students (Wang, Schwartz, et al. 2010). The hypothesis **H_{11}** can be formulated as acculturation stress acting as a mediator between acts as a mediator between diversity experiences and personal development.

The Researchers collectively suggest that acculturation stress can play a mediating role between innovative learning environments and personal development. The investigation found that perceptions of social support mediate the relationship between acculturative stress and internalizing symptoms in immigrant-origin youth (Katsiaficas, Suárez-Orozco et al. 2013). Acculturation stress and social support mediate the relationship between language proficiency and academic, social, and psychological adjustment in international students (Lashari, Awang-Hashim, et al. 2023). There is a negative correlation between acculturation stressors and academic adjustment among Nepalese students in South Korean higher education institutions (Atteraya 2021). Acculturative stress is more common among international students in China, with different levels reported by students from different regions and with different preparation levels (Yu, Chen et al. 2014). The hypothesis **H_{12}** can be formulated as acculturation stress acting as a mediator between innovative learning environments and personal development.

The studies collectively suggest that Institutional Support (IS) can play a crucial role in moderating the relationship between diversity experiences, language proficiency, acculturation stress, and personal development. The quality of cross-racial interactions and students' perceptions of their campus climate influenced the benefits associated with diversity experiences (Denson and Chang 2015). Another investigation highlighted how different

institutional approaches to diversity shaped students' perspectives and approaches to multiculturalism (Warikoo and Deckman 2014). The importance of both diversity and internationalization in creating diverse learning environments that prepare individuals to engage with others who are different from themselves (Wong 2007). The importance of institutional support for diversity in preservice teacher education (Rowan, Brownlee, et al. 2019) The challenges faced by institutions in implementing and deploying technology infrastructure to support learning. This investigation emphasizes the need for collective effort and a mindset shift within institutions to create a universal personal learning environment (White and Davis 2013). The study focuses on professional development for innovative teaching and learning, highlighting the importance of aligning teaching and research to enhance innovation and quality (Isabirye and Moloi 2013). The integration of personal and institutional learning environments, emphasizes the need for interoperability and the coexistence of both types of environments (Conde, García-Peñalvo et al. 2014).

The hypotheses $\mathbf{H_{13}}$, $\mathbf{H_{14}}$, $\mathbf{H_{15}}$, and $\mathbf{H_{16}}$ can be formulated as institutional support moderating the relationship between innovative learning environments and personal development, language proficiency and personal development, acculturation stress and personal development, respectively.

Theoretical Framework

This study aims to investigate the relationship between diversity experiences, and innovative learning environments, and both influence the personal development of international students in China. The theoretical framework integrates many perspectives to analyze the multifaceted relationship of cultural and educational factors shaping the development of students.

Experiential Learning Theory (Kolb 2014), it is highlights in the framework how students actively engage with diversity experiences and innovative learning environments, and also the continuous process of learning and personal development. It also provided an understanding of these experiences and outlined students' thoughtful observation, intellectual conceptualization, active experimentation, and concrete experience.

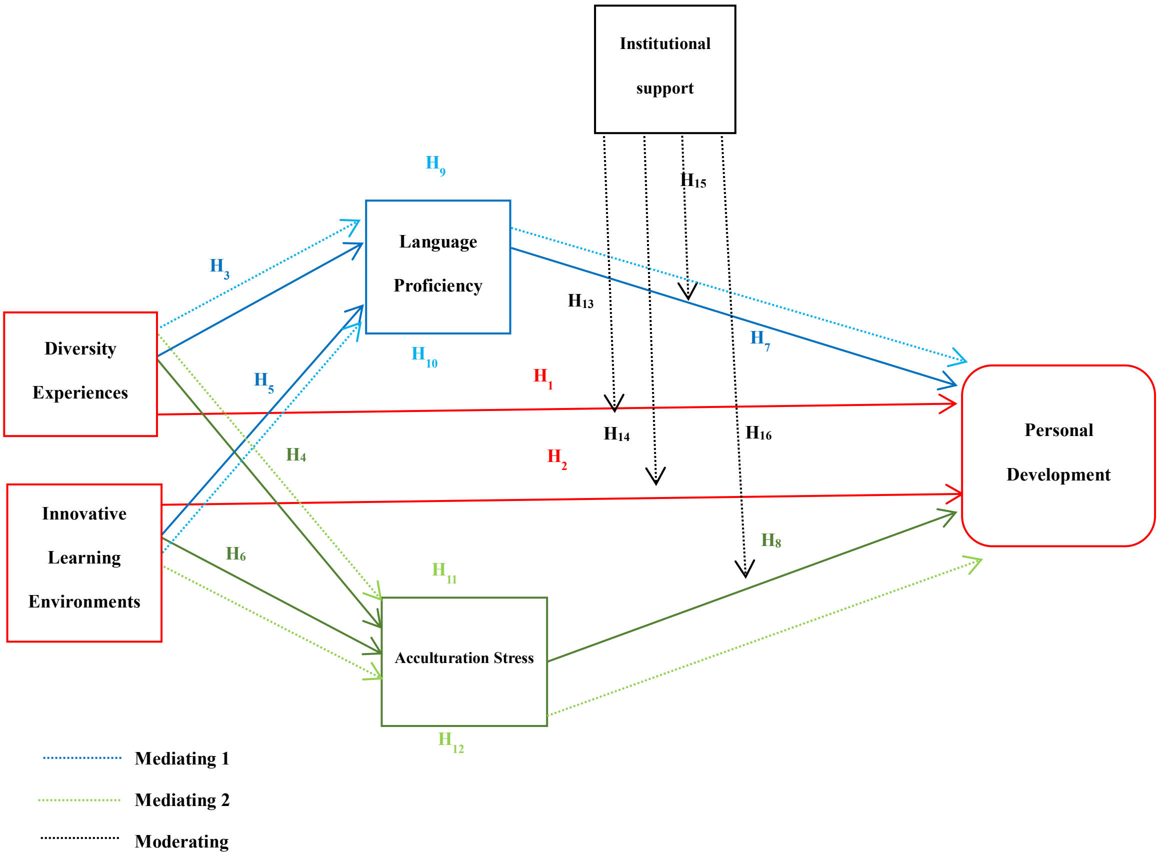

Figure 1: Framework of the study.

Additionally, social Identity Theory (Tajfel and Turner 2004), is demonstrated in the framework that how individuals classify themselves based on cultural attributes, influencing their personal development. This theory provides an understanding of how diversity experiences impact students' social identity and, consequently, their personal growth within the context of innovative learning environments.

Besides, acculturation Theory (Berry 1997), supports the framework, and investigates the mediating role of language proficiency and acculturation stress. It also provides an understanding of the psychological and sociocultural changes students undergo as they navigate diverse educational settings, influencing language development and determining the stressors that affect personal development.

Finally, cultural Dimensions Theory (Hofstede 1984), provides support for individualism-collectivism, power distance, uncertainty avoidance, and masculinity-femininity, and serves to understand the cultural importance in diverse learning environments. It explores how cultural dimensions influence the manifestation of the student's personal development.

The objective of the study is to offer an extensive knowledge of the framework that determines the relationship between student personal development and diversity experiences, language competency, acculturation stress, innovative learning environments, and institutional support.

Research Methodology

With a focus on the mediating roles of language proficiency, acculturation stress, and the moderating role of institutional support in Higher Education Institutions (HEIs), the ongoing research explores the complex interactions between diversity experiences and innovative learning environments and students' personal development in Shaanxi, China. The present study's research approach is explained as follows:

The study uses a quantitative research design, which makes it possible to gather and examine numerical data. This strategy offers an organized way to comprehend the complex interactions between diversity experiences, innovative learning environments, language proficiency, acculturation stress, and institutional support. It is employed convenience sampling

in this investigation to select participants (international students) from HEIs of Shaanxi, China. The sample size is set at 364.

Table 1: Factor loading of study variables.

Variables	Acculturation Stress	Diversity Experience	Innovative Learning Environment	Institutional Support	Language Proficiency	Personal Development
AS 1	0.910					
AS 2	0.887					
AS 3	0.846					
AS 4	0.806					
AS 5	0.814					
DE 1		0.742				
DE 2		0.816				
DE 3		0.793				
DE 4		0.826				
DE 5		0.780				
DE 6		0.731				
DE 7		0.684				
ILE 1			0.873			
ILE 2			0.897			
ILE 3			0.897			
ILE 4			0.913			
ILE 5			0.885			
IS 1				0.881		
IS 2				0.894		
IS 3				0.891		
IS 4				0.822		
IS 5				0.843		
LP 1					0.817	
LP 2					0.800	
LP 3					0.851	
LP 4					0.744	
LP 5					0.848	
PD 1						0.705
PD 2						0.761
PD 3						0.841
PD 4						0.862
PD 5						0.841
PD 6						0.822

This paper uses an adapted survey questionnaire for data collection. The questionnaire

includes two sections: one for gathering demographic information and the other section employs a five-point Likert scale (1=SD to 5=SA) to measure key constructs, including diversity experiences (Bowman and Brandenberger 2012, MEI, SI et al. 2018), innovative learning environments (Boersma, Ten Dam, et al. 2016), language proficiency (Lashari, Awang-Hashim, et al. 2023), acculturation stress (Sandhu and Asrabadi 1994), institutional support (Mason, Morris et al. 2021) and Personal development (Van Staden 2009, Anjum 2020), established from relevant studies.

To gather insights and perspectives comprehensively, participants utilized the Likert scale to express their levels of agreement or disagreement with the provided statements. Smart PLS4 is used for statistical analysis. The relationship between diversity experiences, innovative learning environments, language proficiency, acculturation stress, and institutional support is examined using structural equation modeling (SEM).

Informed agreement is obtained, and participants are kept fully informed about the goal and methods of the study. Participant's confidentiality and anonymity are respected. To make sure the research complies with ethical norms, ethical permission is requested from the appropriate institutional review boards.

Results and Discussions

This section presents the findings of the study and interprets their significance. The results are analyzed to understand the relationships between the key variables.

Demographic analysis

The demographic analysis of the study is carried out by employing 18 questions that are provided in Appendix A. These questions cover topics such as age, gender, marital status,

education, experience, and such (see Appendix A).

The statistics in Table 2 suggest that the constructs measuring have high internal consistency (as indicated by Cronbach's α, ρ_a, and ρ_c) and that they explain a significant portion of the variance in their respective items (as indicated by AVE). This is generally a positive sign of the reliability and validity of the measurement instruments used in the study. It implies that the constructs are well-defined and can be trusted as reliable measures for the study.

Table 2: Construction of Reliability and Validity of factors.

	Cronbach α	ρ_a	ρ_c	Average Variance Extracted
Acculturation Stress	0.909	0.968	0.930	0.728
Diversity Experience	0.867	0.880	0.899	0.564
Innovative Learning Environments	0.937	0.938	0.952	0.798
Institutional Support	0.917	0.919	0.938	0.751
Language Proficiency	0.871	0.871	0.907	0.661
Personal Development	0.892	0.894	0.918	0.651

Table 3 displays various fit indices obtained from a structural equation modeling investigation. These fit indices are essential for assessing how well the model aligns with data. Both models provide a good model fit, with the SRMR (Standardized Root Mean Residual) values lying well below the suggested threshold of 0.08. Both of the models' NFI (Normed Fit Index) values are above the suggested threshold of 0.9, suggesting a satisfactory fit. On the other hand, the first model appears to match the data slightly better, as indicated by its slightly higher NFI value.

Table 3: Various fitness indices obtained from a structural equation modeling investigation.

	Saturated model	Estimated model
Standardized Root Mean Residual	0.058	0.062
d_ULS	1.876	2.190
d_G	0.677	0.696
Chi-square	1407.040	1418.113

Normed Fit Index	0.849	0.848

In Table 4, the Fornell-Larcker criterion table shows the measurement model has good discriminant validity, as each construct is distinct and explains more variance in its measured variables than it shares with other constructs. This is an important finding in SEM as it ensures that the constructs are meaningfully different from each other.

Table 4: Fornell-Larcker criterion.

Variables	AS	DE	ILE	IS	LP	PD
Acculturation Stress	0.853					
Diversity Experience	0.238	0.751				
Innovative Learning Environment	0.260	0.664	0.893			
Institutional Support	0.316	0.640	0.778	0.867		
Language Proficiency	0.282	0.685	0.603	0.620	0.813	
Personal Development	0.253	0.735	0.672	0.730	0.705	0.807

In Table 5 the relationship between diversity experience and personal development is statistically significant ($\beta = 0.267$, $p = 0.000$). The beta coefficient (β) of 0.267 suggests that a 1-unit change in diversity experience is expected to bring about a 26.7% change in personal development, which supports hypothesis H_1. The relationship between innovative learning environment and personal development is not statistically significant ($\beta = 0.098$, $p = 0.140$), thus these results do not support hypothesis H_2. The relationship between diversity experience and language proficiency is highly statistically significant ($\beta = 0.508$, $p = 0.000$). With a beta coefficient (β) of 0.508, a 1-unit change in diversity experience corresponds to a 50.8% change in Language Proficiency, confirming hypothesis H_3. The relationship between diversity experience and acculturation Stress is not statistically significant ($\beta = 0.118$, $p = 0.098$). Hence, results not support hypothesis H_4. The relationship between innovative learning environment and language proficiency is statistically significant ($\beta = 0.266$, $p = 0.000$). The beta coefficient (β) of 0.266 suggests that a 1-unit change in the innovative learning environment is expected to

bring about a 26.6% change in Language Proficiency, providing support for hypothesis H_5. The relationship between innovative learning environment and Acculturation Stress is highly statistically significant (β = 0.182, p = 0.014). This implies that a 1-unit change in the innovative learning environment is associated with an 18.2% change in Acculturation Stress, supporting hypothesis H_6. The relationship between language proficiency and personal development is statistically significant ($\beta = -0.210, p = 0.000$), therefore, hypothesis H_7 is supported. The relationship between acculturation stress and personal development is statistically not significant ($\beta = -0.2036, p = 0.310$). These results do not support hypothesis H_8.

Table 5: Path Coefficients Analyses Revealing Relationships between Variables.

	β	STDEV	t- statistics	p-values
**DE→ PD**	0.267	0.064	4.165	0.000
**ILE → PD**	0.098	0.066	1.477	0.140
**DE → LP**	0.508	0.061	8.336	0.000
**DE → AS**	0.118	0.071	1.655	0.098
**ILE → LP**	0.266	0.068	3.918	0.000
**ILE → AS**	0.182	0.074	2.449	0.014
**ILE → PD**	0.210	0.052	4.061	0.000
**AS → PD**	-0.036	0.036	1.016	0.310
**IS → PD**	0.373	0.062	6.025	0.000
**IS * LP → PD**	-0.081	0.055	1.474	0.141
**IS * DE → PD**	-0.097	0.064	1.514	0.130
**IS * ILE → PD**	0.140	0.059	2.392	0.017
**IS * AS → PD**	0.036	0.039	0.928	0.353

In Table 6 the specific indirect effect of diversity experience on personal development through language proficiency is 0.107. The T-statistic is 3.689, indicating that the relationship is highly statistically significant (p = 0.000). This suggests that there is a significant indirect effect of diversity experience on personal development through language proficiency, hence H_9 is approved. Furthermore, the specific indirect effect of an innovative learning environment on personal development through language proficiency is 0.056. The T-statistic is 2.782, indicating

that the relationship is statistically significant (p = 0.005).

Table 6: Specific Indirect Effects

Variable Relationship	β	Standard deviation	t- statistics	p-values
**DE → LP → PD**	0.107	0.029	3.689	0.000
**ILE → LP → PD**	0.056	0.020	2.782	0.005
**DE -> AS → PD**	-0.004	0.006	0.753	0.452
**ILE → AS → PD**	-0.007	0.008	0.882	0.378

This suggests that there is a significant indirect effect of an innovative learning environment on personal development through language proficiency, thus H_{10} is approved here. Moreover, the specific indirect effect of diversity experience on personal development through acculturation stress is -0.004. The T-statistic is 0.753, indicating that the relationship is not statistically significant (p = 0.452). This suggests that the indirect effect of diversity experience on personal development through acculturation stress is not significant in this analysis, so H_{11} is not approved. Lastly, the specific indirect effect of an innovative learning environment on personal development through acculturation stress is -0.007.

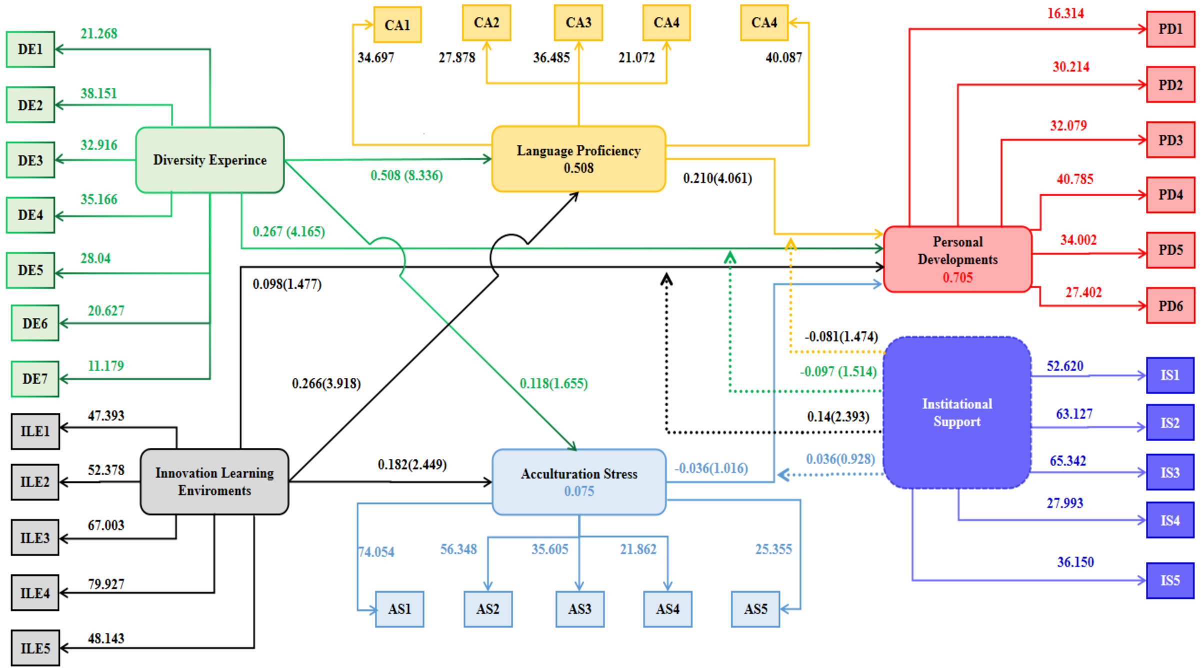

Figure 2: The bootstrapping analysis of the variables of the study.

The T-statistic is 0.882, which indicates that the relationship is not statistically significant ($p = 0.378$). This suggests that the indirect effect of an innovative learning environment on personal development through acculturation stress is not significant in this analysis, hence H_{12} is also not approved. Figure 3 explains that institutional support strengthens the relationship between the diversity experience and personal development, so H_{13} is approved. However, the analysis results do not show any moderation effect of institutional support on the relationship between innovative learning environments and personal development. Therefore, H_{14} is rejected based on the information provided. Similarly, results also do not show an y moderation effect of institutional support on the relationship between language proficiency and personal development. Consequently, H_{15} is rejected. Lastly, results also do not show any moderation effect of institutional support on the relationship between acculturation stress and personal development. Hence H_{16} is rejected.

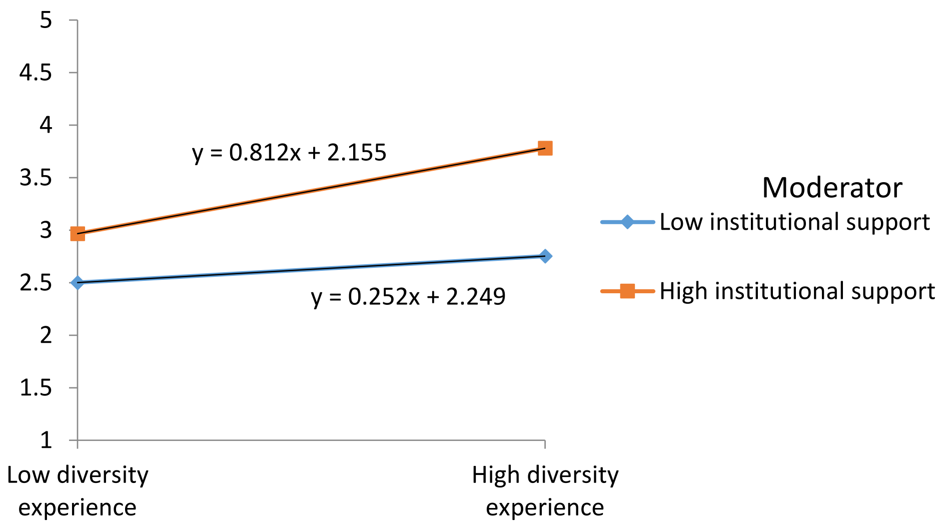

Figure 3: The moderating effect on personal development.

Discussion

The results of this study, demonstrating a statistically significant positive correlation

between diversity experiences and personal development, are consistent with the outcomes of Jenert and Brahm (2021), who emphasized the interaction between contextual and personal diversity and the role that individual features serve in shaping students' experiences in higher education. Furthermore, the results of a study by Krykunova, Pashchenko et al. (2023) suggested that although innovative learning environments may enhance learning outcomes, their impact on personal development might be indirect or mediated by other factors, are consistent with our observation that there is no significant immediate connection between learning environments and personal development.

The research indicates that diversity experiences and language proficiency are legitimate measures, and it is linked to the findings of Scholl, Fontes et al. (2021), that persons may effectively assess their skills. The analysis shows a high relationship between diversity experience and language proficiency.

Furthermore, a study by Bae (2020) found that bicultural identity possessed a positive effect on psychological well-being and a negative impact on acculturative stress, with the second mediating the former. The results of this study are consistent with the non-significant relationship between diversity experience and acculturation stress. According to Köroğlu (2020) and Borthwick and Plutino (2020), there are noteworthy correlations between innovative learning environments and language proficiency as well as acculturation stress. These findings demonstrate the potential of technology-enhanced settings in language assessment and fluent interpersonal interaction, respectively. Moreover, the interaction between acculturation stress and language proficiency with personal development is significant. This is consistent with previous studies by Taušová, Bender et al. (2019), and Lashari, Awang-Hashim et al. (2023),

who found that language proficiency promotes people's personal development.

Language proficiency exhibits indirect substantial effects of diversity experience and innovative learning scenarios on personal development, which falls in line with the research findings of Taušová, Bender et al. (2019) and Gluchmanova (2019). Such findings illustrate the importance that language proficiency serves in connecting different situations and innovative learning settings alongside individual development. The investigation illustrates that institutional support, including actions like diversity experience and inclusive teaching, can significantly boost the relationship between diversity experiences and personal development. Finally, there is a greater impact of institutional support on the association between diversity experiences and personal development, which is consistent with the investigation of Cabler (2019).

In general, it highlights the relationships that are important in influencing the personal development of international students between diversity experiences, innovative learning environments, language proficiency, acculturation stress, and institutional support. The necessity of a complete approach to promote complete student growth is highlighted by the combined effect of these effects, according to the statement. How these elements interact shows how important it is to recognize the complex ways in which educational environments benefit students as they learn.

Conclusions

this investigation has elucidated the complex relationships between diversity experiences, innovative learning environments, language proficiency, acculturation stress, and institutional support, and their impact on the personal development of international students. The findings

underscore the significance of diversity experiences and language proficiency as predictors of personal development, while innovative learning environments and institutional support play a crucial mediating role in these relationships. Moreover, the results highlight the importance of addressing acculturation stress, which can have a negative impact on personal development.

The study's outcomes are consonant with extant research, emphasizing the interconnectedness of these factors and the need for a comprehensive approach to foster holistic student growth. By recognizing the complex ways in which educational environments influence student development, educators and policymakers can work towards creating inclusive and supportive learning environments that provide to the diverse needs of international students. This study contributes to the ongoing academic discussion on the importance of promoting personal development in higher education, underscoring the critical role of diversity experiences, language proficiency, and institutional support in shaping the experiences of international students.

References

Abu-Kaf, S. and E. Khalaf (2020). "Acculturative stress among Arab students in Israel: the roles of sense of coherence and coping strategies." International Journal of Environmental Research and Public Health **17**(14): 5106.

Ahn, L. H., D. M. Kivlighan Jr and C. E. Hill (2022). "Helping skills courses: The effects of student diversity and numeric marginalization on counseling self-efficacy, counseling self-stigma, and mental health." Journal of counseling psychology **69**(1): 27.

Anjum, S. (2020). "Impact of internship programs on professional and personal development of business students: a case study from Pakistan." Future Business Journal **6**(1): 2.

Ariyan, M. and T. Vadeeva (2019). "Adult learners' personal development by means of foreign language teaching: aims and ways of implementation." Language and Culture(46): 184-202.

Atteraya, M. S. (2021). "Acculturation stressors and academic adjustment among Nepalese students in South Korean higher education institutions." International Journal of Environmental Research and Public Health **18**(12): 6529.

Bae, S. M. (2020). "The relationship between bicultural identity, acculturative stress, and psychological well-being in multicultural adolescents: Verification using multivariate latent growth modelling." Stress and Health **36**(1): 51-58.

Banditvilai, C. (2016). "Enhancing students language skills through blended learning." Electronic Journal of e-Learning **14**(3): pp223-232-pp223-232.
Bernard, R. M., E. Borokhovski, R. F. Schmid, D. I. Waddington and D. I. Pickup (2019). "Twenty-first century adaptive teaching and individualized learning operationalized as specific blends of student-centered instructional events: A systematic review and meta-analysis." Campbell Systematic Reviews **15**(1-2).
Berry, J. W. (1997). "Immigration, acculturation, and adaptation." Applied psychology **46**(1): 5-34.
Boersma, A., G. Ten Dam, W. Wardekker and M. Volman (2016). "Designing innovative learning environments to foster communities of learners for students in initial vocational education." Learning Environments Research **19**: 107-131.
Borthwick, K. and A. Plutino (2020). Education 4.0 revolution: transformative approaches to language teaching and learning, assessment and campus design, Research-publishing. net.
Bourgeois-Bougrine, S., S. Latorre and F. Mourey (2018). "Promoting creative imagination of non-expressed needs: exploring a combined approach to enhance design thinking." Creativity Studies **11**(2): 377-394.
Bowman, N. A. and J. W. Brandenberger (2012). "Experiencing the unexpected: Toward a model of college diversity experiences and attitude change." The Review of Higher Education **35**(2): 179-205.
Bruguier, L. R. and L. M. Greathouse Amador (2012). "New educational environments aimed at developing intercultural understanding while reinforcing the use of English in experience-based learning." Profile Issues in TeachersProfessional Development **14**(2): 195-211.
Cabler, K. (2019). "Exploring the impact of diversity training on the development and application of cultural competence skills in higher education professionals."
Cao, C., D. C. Zhu and Q. Meng (2016). "An exploratory study of inter-relationships of acculturative stressors among Chinese students from six European union (EU) countries." International journal of intercultural relations **55**: 8-19.
Chan, S. and J. M. KAPONG (2021). "PERSONAL GROWTH THROUGH ENGLISH PROFICIENCY: A CASE STUDY IN SARAWAK." A. Âsaui atyndaġy Halyķaralyķ ķazaķ-tùrìk universitetìnìņ habaršysy(122): 85-93.
Charbonneau, S., J. deLeyer-Tiarks, L. C. Caterino and M. Bray (2022). "A meta-analysis of school-based interventions for student refugees, migrants, and immigrants." Journal of Prevention & Intervention in the Community **50**(4): 434-449.
Conde, M. Á., F. García-Peñalvo, M. Alier, E. Mayol and C. Fernández-Llamas (2014). "Implementation and design of a service-based framework to integrate personal and institutional learning environments." Science of Computer Programming **88**: 41-53.
Crockett, L. J., M. I. Iturbide, R. A. Torres Stone, M. McGinley, M. Raffaelli and G. Carlo (2007). "Acculturative stress, social support, and coping: relations to psychological adjustment among Mexican American college students." Cultural Diversity and Ethnic Minority Psychology **13**(4): 347.
De Roover, K., J. K. Vermunt and E. Ceulemans (2022). "Mixture multigroup factor analysis for unraveling factor loading noninvariance across many groups." Psychological Methods **27**(3): 281.
Denson, N. and M. J. Chang (2015). "Dynamic relationships: Identifying moderators that maximize benefits associated with diversity." The Journal of Higher Education **86**(1): 1-37.

Dilara, Ö. and H. A. YILMAZ (2021). "Educational journey of refugee students: Suggestions for practitioners." Turkish Online Journal of Qualitative Inquiry **12**(2): 405-437.
do Amaral, C. C. B., M. Romani-Dias and S. Walchhutter (2022). "International Brazilian students: Motivators, barriers, and facilitators in higher education." SAGE Open **12**(1): 21582440221088022.
Edara, I. R. (2018). "Social and spiritual dimensions as protective factors in the relationship between acculturative stress and subjective well-being among international students in Taiwan." Psychology **9**(07): 1582.
Egarievwe, S. U. (2021). Development of Active-Learning Units in Nuclear Engineering. EPJ Web of Conferences, EDP Sciences.
Faez, F. and M. Karas (2017). "Connecting language proficiency to (self-reported) teaching ability: A review and analysis of research." RELC journal **48**(1): 135-151.
Fahrutdinova, R. A., I. E. Yarmakeev and R. R. Fakhrutdinov (2014). "The Formation of Students' Foreign Language Communicative Competence during the Learning Process of the English Language through Interactive Learning Technologies (The Study on the Basis of Kazan Federal University)." English Language Teaching **7**(12): 36-46.
Gabryś-Barker, D. (2019). "Cognitive and affective dimensions of foreign language learning invironments: a Polish-Turkish comparative study." Neofilolog(52).
Gebregergis, W. T. (2018). "Major causes of acculturative stress and their relations with sociodemographic factors and depression among international students." Open Journal of Social Sciences **6**(10): 68-87.
Geeraert, N. and S. Demoulin (2013). "Acculturative stress or resilience? A longitudinal multilevel analysis of sojourners' stress and self-esteem." Journal of Cross-Cultural Psychology **44**(8): 1241-1262.
Genkova, P. (2016). "Experience abroad and its relation to intercultural competence and cross-cultural tolerance." International Journal of Business and Management **11**(5): 1.
Glomo-Narzoles, D. T. and D. Glomo-Palermo (2021). "Examining the Academic and Non-Academic Correlates of Student Retention." International Journal of Publication and Social Studies **6**(1): 34-41.
Gluchmanova, M. (2019). "Innovative Methods of Education for Development of Language Skills." NORDSCI.
Herzog, S. (2022). "Classroom diversity and college student dropout: New evidence from panel data and objective measures." Innovative Higher Education **47**(4): 609-637.
Hofstede, G. (1984). Culture's consequences: International differences in work-related values, sage.
Howard, J. and A. Scott (2017). "Any time, any place, flexible pace: Technology-enhanced language learning in a teacher education programme." Australian Journal of Teacher Education **42**(6): 51-68.
Isabirye, A. and K. Moloi (2013). "Professional development and its implications for innovative teaching and learning in one South African higher education institution." Mediterranean Journal of Social Sciences **4**(14): 101.
Jenert, T. and T. Brahm (2021). "The Interplay of Personal and Contextual Diversity during the First Year at Higher Education: Combining a Quantitative and a Qualitative Approach." Frontline Learning Research **9**(2): 50-77.
Jo, D., S. Woo and E. Yang (2023). "Role of family acculturation stress in career development

among adolescents from multicultural families in Korea." Journal of Career Development **50**(4): 901-916.
Katsiaficas, D., C. Suárez-Orozco, S. R. Sirin and T. Gupta (2013). "Mediators of the relationship between acculturative stress and internalization symptoms for immigrant origin youth." Cultural Diversity and Ethnic Minority Psychology **19**(1): 27.
Keengwe, J. (2010). "Fostering cross cultural competence in preservice teachers through multicultural education experiences." Early Childhood Education Journal **38**: 197-204.
Killian, T. and M. Floren (2020). "Exploring the relationship between pedagogy and counselor trainees' multicultural and social justice competence." Journal of Counseling & Development **98**(3): 295-307.
Kim, J., E. Malonebeach, J. Heo, J. Kim and M. Kim (2015). "Exploratory examination of the relationship between acculturation and personal growth among Korean immigrants." Psychological Reports **116**(2): 613-630.
Kolb, D. A. (2014). Experiential learning: Experience as the source of learning and development, FT press.
Köroğlu, Z. Ç. (2020). Assessing Language Learning in Technology-Enhanced Environments. ICT-Based Assessment, Methods, and Programs in Tertiary Education, IGI Global**:** 45-64.
Koschollek, C., K. Kajikhina, S. Bartig, M.-L. Zeisler, P. Schmich, A. Gößwald, A. Rommel, T. Ziese and C. Hövener (2022). "Results and strategies for a diversity-oriented public health monitoring in Germany." International Journal of Environmental Research and Public Health **19**(2): 798.
Krykunova, O., M. Pashchenko and O. Sobolenko (2023). THE ROLE OF INNOVATIVE LEARNING TECHNOLOGIES IN FORMING THE CREATIVE PERSONALITY OF STUDENTS. SWorld-Ger Conference proceedings.
Kubota, R., T. Austin and Y. Saito-Abbott (2003). "Diversity and inclusion of sociopolitical issues in foreign language classrooms: An exploratory survey." Foreign Language Annals **36**(1): 12-24.
Lashari, S. A., R. Awang-Hashim, T. A. Lashari and A. Kaur (2023). "Acculturation stress and social support for international students' adjustment in Malaysia: does language proficiency matter?" Journal of Applied Research in Higher Education **15**(2): 496-508.
Law, W. and S. Liu (2023). "Basic Need Satisfaction Intervention for Mainland Chinese International Students' Adjustment to College." Journal of Studies in International Education **27**(1): 100-118.
Lintunen, P., M. Mutta and S. Pelttari (2017). "Profiling language learners in hybrid learning contexts: Learners' perceptions." The EuroCALL Review **25**(1): 61-75.
Machul, M., M. Bieniak, J. Chałdaś-Majdańska, J. Bąk, A. Chrzan-Rodak, P. Mazurek, P. Pawłowski, D. Makuch-Kuśmierz, A. Obuchowska and A. Bartoszek (2020). "Lifestyle practices, satisfaction with life and the level of perceived stress of Polish and foreign medical students studying in Poland." International Journal of Environmental Research and Public Health **17**(12): 4445.
Mangus, L., C. Somers, J. Yoon, T. Partridge and F. Pernice (2021). "Examination of college student achievement within an ecological framework." Journal of Adult and Continuing Education **27**(2): 231-247.
Marina, I., B. Nonna and I. Vitaly (2018). Discussion on some features of innovative system of professional training. 5th International Conference on Education, Language, Art and Inter-

cultural Communication (ICELAIC 2018), Atlantis Press.
Mason, S., J. E. Morris and M. K. Merga (2021). "Institutional and supervisory support for the Thesis by Publication." Australian Journal of Education **65**(1): 55-72.
MEI, H., R.-y. SI and J. WANG (2018). "On the Relationship Between College Students' Diversity Experiences and Their Critical Thinking Disposition." Journal of Northeastern University (Social Science) **20**(4): 412.
Melo, C. O., M. C. M. S. Luft and R. O. Rocha (2021). "Influencing elements of technological adoption: Case study about management in an educational institution." Contextus–Revista Contemporânea de Economia e Gestão **19**: 124-144.
Moeller, A. K. and T. Catalano (2015). "Foreign language teaching and learning."
Murray, N. (2016). "Dealing with diversity in higher education: awareness-raising and a linguistic perspective on teachers' intercultural competence." International Journal for Academic Development **21**(3): 166-177.
Novita, D. and O. Purwati (2021). "Incorporating culture and textbook adaptation practice for promoting intercultural competence in ELT: teachers' perspectives." JEES (Journal of English Educators Society) **6**(1): 96-104.
Onosu, G. (2021). "The impact of cultural immersion experience on identity transformation process." International Journal of Environmental Research and Public Health **18**(5): 2680.
Orellana, M. F., D. C. Martínez, C. H. Lee and E. Montaño (2012). "Language as a tool in diverse forms of learning." Linguistics and Education **23**(4): 373-387.
Parkhouse, H., C. Y. Lu and V. R. Massaro (2019). "Multicultural education professional development: A review of the literature." Review of Educational Research **89**(3): 416-458.
Poyrazli, S., P. R. Kavanaugh, A. Baker and N. Al-Timimi (2004). "Social support and demographic correlates of acculturative stress in international students." Journal of College Counseling **7**(1): 73-82.
Ramdhonee, K. and U. Bhowon (2012). "Acculturation strategies, personality traits and acculturation stress: A study of first generation immigrants from transnational marital context." Psychology and Developing Societies **24**(2): 125-143.
Rowan, L., J. L. Brownlee and M. Ryan (2019). Teaching teachers: what [should] teacher educators "know" and "do" and how and why it matters, Taylor & Francis. **47:** 210-215.
Sancar, R., D. Atal and D. Deryakulu (2021). "A new framework for teachers' professional development." Teaching and Teacher Education **101**: 103305.
Sandhu, D. S. and B. R. Asrabadi (1994). "Development of an acculturative stress scale for international students: Preliminary findings." Psychological reports **75**(1): 435-448.
Scholl, A. P., A. B. A. d. L. Fontes and I. Finger (2021). "Can bilinguals rate their proficiency accurately in a language background questionnaire?: a correlation between self-rated and objective proficiency measures." Revista da ANPOLL. Florianópolis, SC. Vol. 52, n. 1 (jan./maio 2021), p.[142]-161.
Shaqour, E. N. (2021). "Using Modern Teaching Strategies to Improve Architectural Design Studio Pedagogy in West Bank." MEJ. Mansoura Engineering Journal **46**(1): 46-53.
Simons, L., S. Schoneman, M. Hoffman, K. Beller and N. Blank (2023). "Reframing Cultural Competence and Civic Engagement: A Replication Study of High Impact Practices in Undergraduate Psychology Courses." European Journal of Education Studies **10**(2).
Souto-Manning, M., B. Falk, D. López, L. Barros Cruz, N. Bradt, N. Cardwell, N. McGowan, A. Perez, A. Rabadi-Raol and E. Rollins (2019). "A transdisciplinary approach to equitable

teaching in early childhood education." Review of Research in Education **43**(1): 249-276.
Stoszkowski, J. R. and L. McCarthy (2018). "Students' perceptions of the learner attributes required for (and resulting from) heutagogical learning." Journal of Learning Development in Higher Education **14**.
Sullivan, C. and S. Kashubeck-West (2015). "The interplay of international students' acculturative stress, social support, and acculturation modes." Journal of International Students **5**(1): 1-11.
Supeni, S. and A. Fauziah (2020). "Aligning the TOEFL prediction scores to the Common European Framework of Reference for languages (CEFR) level." JELL (Journal of English Language and Literature) STIBA-IEC Jakarta **5**(01): 33-42.
Tajfel, H. and J. C. Turner (2004). The social identity theory of intergroup behavior. Political psychology, Psychology Press**:** 276-293.
Taušová, J., M. Bender, R. Dimitrova and F. van de Vijver (2019). "The role of perceived cultural distance, personal growth initiative, language proficiencies, and tridimensional acculturation orientations for psychological adjustment among international students." International Journal of Intercultural Relations **69**: 11-23.
Tawafak, R., S. Malik, R. Mathew, M. Ashfaque, J. Jabbar, M. AlNuaimi, A. ElDow and G. Alfarsi (2021). "A combined model for continuous intention to use e-learning system."
Üzar-Özçetin, Y. S., S. Çelik and G. Özenç-Ira (2022). "The relationships among psychological resilience, intercultural sensitivity and empathetic tendency among teachers of Syrian refugee children in Turkey." Health & Social Care in the Community **30**(2): e410-e419.
van den Berg, F., J. Brouwer, H. Loerts, R. Knooihuizen and M. Keijzer (2020). Foreign language learning to promote cognitive flexibility and well-being in healthy seniors and those with Mild Cognitive Impairment. Bilingualism and the Brain.
Van Staden, A. (2009). The Balanced Scorecard as performance measurement and personal development tool in a steel organisation, North-West University.
Wang, S. C., S. J. Schwartz and B. L. Zamboanga (2010). "Acculturative stress among Cuban American college students: Exploring the mediating pathways between acculturation and psychosocial functioning." Journal of applied social psychology **40**(11): 2862-2887.
Warikoo, N. K. and S. L. Deckman (2014). Beyond the numbers: Institutional influences on experiences with diversity on elite college campuses. Sociological Forum, Wiley Online Library.
White, S. and H. C. Davis (2013). Making it rich and personal: crafting an institutional personal learning environment. Technologies, innovation, and change in personal and virtual learning environments, IGI Global**:** 177-192.
Wong, M. S. (2007). Supporting Diversity and Internationalization through Transformative Learning Experiences. Forum on Public Policy Online, ERIC.
Yamat, H. (2012). "Developing identity in diversity: A second language acquisition experience." GEMA Online Journal of Language Studies **12**(2).
Yang, F. (2020). "Influence of Chinese International Students' Intercultural Communicative Competence to Their English Proficiency in the US." US-China Education Review **10**(2): 61-78.
Yu, B., X. Chen, S. Li, Y. Liu, A. J. Jacques-Tiura and H. Yan (2014). "Acculturative stress and influential factors among international students in China: A structural dynamic perspective." PloS one **9**(4): e96322.
Yulianti, S. A. and M. Z. Badruzsaufari (2020). "Learning Tools Development in Waste Topic

on Students Critical Thinking Skills of Vocational School."
Zhou, M. Y. (2015). Diversity and teacher education: Cultural and linguistic competency for teachers. Handbook of research on cross-cultural approaches to language and literacy development, IGI Global: 299-320.
Zylkiewicz-Plonska, E. and E. Acienė (2014). "The need for creating and developing intercultural competences among international students." Tiltai: socialiniai mokslai(2): 89-107.

Other

Click here to access/download
Supporting Information
supplementary Materials.docx
