# Supplementary material for: The influence of language proficiency, acculturation stress, and institutional support in enhancing personal development of international students in China
Source: PLoS One. 2025 Apr 4;20(4):e0315040. doi: 10.1371/journal.pone.0315040 (PMC11970667; doi:10.1371/journal.pone.0315040)
Supplement: Attachment — Submitted filename: Review Report for Exploring the Relationship of Di.docx [file pone.0315040.s005.docx]

Review Report for **Exploring the Relationship of Diversity Experiences, Innovative Learning Environments, and Personal Development among International Students in China**

The paper sounds good and interesting, yet the following are meant to enrich it:

1. The first finding stated in the abstract reveals the existence of a positive association between experience and personal development while the first sentence in the abstract focuses on the association between experience and innovative learning environment and their impact on personal development. Do the two sentences mean the same? If yes, please re-consider the introductory statement of the abstract.
2. Some recommendations or pedagogical implications are preferable to end up the abstract with.
3. The research gap and significance are not clearly stated in the abstract.
4. Referencing the source in “… are emphasized by Souto-Manning, Falk et al. (2019).” is not proper. Only the last name of the first author is used followed by ‘et al’. Follow the APA system in this regard, the same with the other sources in the second and other paragraphs of the Introduction section.
5. In the in-text citation, the ampersand ‘&’ is used to connect two names, while ‘and’ is used in the text only. Correct accordingly.
6. When there is a quotation, a page number is required.
7. Why do abbreviations such as PD, ILE and DE appear in the Hypotheses section? They should appear the first time the words are used in the Introduction section.
8. Editing is highly recommended to fix such language issues as in “… the relationship between diversity experiences and personal development which hypnotized as H1 is exist according to the literature.” and other places.
9. A paragraph should start with the authors’ names as a subject followed by a year between round brackets. Do not start a sentence with a round bracket as in “(Katsiaficas, Suárez-Orozco, et al. 2013) suggested that acculturation stress can play a mediating …”.
10. The hypotheses section is very long and confusing. I suggest grouping the hypotheses after reducing them to 5-6 and under the title ‘Hypotheses’ and grouping the studies under the title ‘Previous Studies’.
11. I suggest having a note below Table 1 referring to the abbreviations included in the table to make it easier for the reader to understand the study variables.
12. After the Introduction and before the Results and Discussions, the researchers need to have two main sections: Literature Review and Methodology.
13. Under the section Results and Discussion, there should be two sub-sections: Results and Discussion.
14. I suggest having the same point raised in 11 above for Table 2.
15. The researchers need to end their paper with recommendations or pedagogical implications.
16. The whole reference list should be re-considered in light of the APA system of documentation.
